# Artificial rearing alters intestinal microbiota and induces inflammatory response in piglets

**DOI:** 10.3389/fmicb.2022.1002738

**Published:** 2022-10-06

**Authors:** Qi Han, Xiaohong Zhang, Haoyang Nian, Honggui Liu, Xiang Li, Runxiang Zhang, Jun Bao

**Affiliations:** ^1^College of Animal Science and Technology, Northeast Agricultural University, Harbin, China; ^2^Key Laboratory of Swine Facilities Engineering, Ministry of Agriculture and Rural Affairs, Harbin, China

**Keywords:** artificial rearing, piglet, diarrhea, microbiota, NF-κB pathway

## Abstract

With the ongoing genetic selection for high prolificacy in sow lines and the improvements in environment and farm management, litter size has increased in recent years. Artificial rearing is becoming widely used to raise the surplus piglets in pig industry. This study aimed to investigate the changes that happened in the morphology, microbiota, mucosal barrier function, and transcriptome caused by artificial rearing in piglet colon. Two hundred and forty newborn piglets were randomly assigned into three treatments, sow rearing until weaning (CON group), artificial rearing from day 21 (AR21 group), and artificial rearing from day 7 (AR7 group). On day 35, the piglets were euthanized to collect colon samples. The results showed that the artificially reared-piglets displayed increased pre-weaning diarrhea incidence and reduced growth performance. Artificial rearing changed the diversity and structure of colonic microbiota and increased relative abundance of harmful bacteria, such as *Escherichia-Shigella*. In addition, the morphological disruption was observed in AR7 group, which was coincided with decreased tight junction proteins and goblet cell numbers. Moreover, the expression of TNFSF11, TNF-α, IL-1β, TLR2, TLR4, MyD88, NF-κB, COX-2, PTGEs, iNOS, IL-2, IL-6, IL-17A, and IFN-γ was upregulated in the colon of the artificially reared-piglets, while the expression of IL-1Ra and IκBα was downregulated, indicating that artificial rearing induced inflammatory response through the activation of NF-κB pathway. Furthermore, artificial rearing regulated SLC family members, which affected solute transport and destroyed intestinal homeostasis. In conclusion, artificial rearing caused microbiota alteration, morphology disruption, the destruction of mucosal barrier function, and inflammatory response, and thus, led to subsequent increased diarrhea incidence and reduced growth performance.

## Introduction

In recent years, genetic selection toward hyper-prolificacy has elevated the number of born piglets per litter, routinely exceeding the number of functional teats (Lanferdini et al., 2018). Unfortunately, larger litter size was linked to lower birth weight and growth rate, which ultimately resulted in higher pre-weaning mortality (Quesnel et al., 2008; Peltoniemi et al., 2021). Artificial rearing is a common practice to improve piglet survival. An investigation found that 31% of pig farmers adopted this strategy to raise the surplus piglets and 85% supplemented formula milk to piglets in the Flemish region of Belgium (Vandenberghe, 2012). A recent study reported the adverse effects of artificial rearing on growth performance, behavior, and emotional state of piglets (Schmitt et al., 2019). In the artificial rearing system, milk replacer can meet the nutritional requirements of piglets to the greatest extent, but still lack multiple functional and bioactive components (Salmon et al., 2009; Levast et al., 2014). Rasmussen et al. (2016) reported that diarrhea frequently occurred in artificially reared-piglets. Vergauwen et al. (2017) found that artificial rearing altered the morphology, permeability, and redox state in the small intestine of piglets. Epidemiological studies reported that formula milk and prolonged duration of enteral feeding led to an increased risk of necrotizing enterocolitis (NEC) in infants (Lucas and Cole, 1990; Berkhout et al., 2018). NEC most commonly affects the proximal ascending colon and the terminal ileum (Di Lorenzo et al., 1995). It could be hypothesized that artificial rearing might mediate intestinal dysfunction in piglets.

Gut microbiota is implicated in a range of host physiological processes, including nutrition absorption, energy utilization, and immune modulation (Nicholson et al., 2012). A stable and diverse gut microbiota is essential for intestinal health and pig growth. The composition of gut microbiota can be affected by many factors, such as diet, age, environment, and antibiotics (Rinninella et al., 2019). The presence of sow feces can influence piglet microbiota development, growth, and survival (Nowland et al., 2021). Piccolo et al. (2017) compared the intestinal microbiota of piglets fed with the dairy or soy infant formulas and found that soy formula feeding resulted in lower community richness in jejunum. The rats fed with formula milk had a lower microbial diversity and decreased acetic acid concentration in cecum (Liu et al., 2016). Underwood et al. (2014) found that formula feeding altered microbiota composition and triggered inflammatory response and mucosal disease in rat ileum. In this study, we are particularly interested in the effects of artificial rearing on the intestinal microbiota of piglets and consequently, on the incidence of diarrhea and growth performance.

Artificial rearing has been widely adopted in the Netherlands, the United States, and increasingly in Germany (ŠPinka, 2018). So far, the scientific evidences regarding the effects of artificial rearing on intestinal health, especially colon remain scanty and require further investigation. Moreover, the pigs share many similarities with humans in anatomy, morphology, and physiology of the gastrointestinal tract (Gonzalez et al., 2015) and have been used with increasing frequency in NEC research (Mendez et al., 2020). In the present study, we established a piglet model of artificial rearing, and then measured growth performance of piglets, observed colon morphology, and analyzed the structural and functional changes of intestinal microbiota. The next-generation sequencing was performed to illustrate the molecular mechanism of colonic dysfunction. Hopefully, our study will give a comprehensive understanding of the pathogenesis of artificial rearing-induced colonic dysfunction and provide a reference for comparative medicine.

## Materials and methods

### Ethics statement

This study was approved by the Institutional Animal Care and Use Committee of Northeast Agricultural University. All procedures were performed in accordance with the EU Directive 2010/63/EU on the protection of animals used for scientific purposes.

### Animals and experimental design

Thirty sows (Duroc × Min) were artificially inseminated with semen sourced from the same Yorkshire boar. Two hundred and forty newborn piglets (Yorkshire × Duroc × Min) were used in this animal experiment. The piglets were randomly divided into three treatments at birth (eight piglets per litter and 10 litters per group), artificial rearing from 7 days of age (AR7 group), artificial rearing from 21 days of age (AR21 group), and sow rearing until weaning (CON group). All piglets were weaned on day 35. During the period of 1–35 days of age, the CON piglets suckled the sows, whereas AR7 and AR21 piglets were fed with milk replacer (Joosten Products B.V., Netherlands) after being moved away from the sows. The milk replacer was made by mixing 150 g of milk replacer powder into 1 L of water at 45°C, which was provided using a milk cup with an inside diameter of 16 cm. The daily intake of milk replacer was 360 ml/kg body weight (BW). The nutrient composition of milk replacer was shown in Supplementary Table 1. Milk cup was cleaned and disinfected once daily. Feces and urine were cleaned twice daily. The piglets with a BW lower than 700 g were classified as runt and excluded from this study.

The individual farrowing crate consisted of a pen (2.0 m × 4.5 m) for sow to lie down, rest, and stand up and a protected area (1.0 m × 2.0 m) for piglets. Each farrowing crate was equipped with a feeder and a nipple drinker for the sows, and a 100 W infrared (IR) lamp, an electric heating plate, and a nipple drinker for the piglets. The ambient temperature was on average 25.7°C (range: min. 24.2°C to max. 27.5°C) and the temperature in protected area was maintained at approximately 30°C. Relative humidity varied from 65 to 75%. Daily photoperiod was 16 h light:8 h darkness.

### Growth performance measurements

All piglets were individually weighed on day 0 and weekly during the experiment. The average daily gain (ADG) was calculated for each week and the entire experimental period.

### Diarrhea incidence

From 7 days of age, the diarrhea episodes were daily recorded. The diarrhea was defined as loose and watery stool or stool attached to the skin around the anus. The diarrhea incidence of each week was calculated as follows:


$$\mathrm{Diarrhea incidence}\;\left(\%\right)=\frac{A}{M}\times 100\%$$

where A = the number of piglets with diarrhea recorded for each week. *M* = Total number of piglets × 7 days.

### Sample collection

On day 35, 10 piglets were randomly selected from each group (one piglet from each litter) and euthanized. The luminal contents of the ascending colon were collected, rapidly frozen in liquid nitrogen, and then stored at −80°C. The colon segments (approximately 1 cm) were fixed in 4% paraformaldehyde for histological examination. After washing with PBS solution twice, the mucosa samples were scraped on an ice bag using sterile glass microscope slides. Then, the samples were rapidly frozen in liquid nitrogen and stored at −80°C for further analysis.

### 16S rDNA sequencing

16S rDNA sequencing was conducted on 10 biological replicates in each group. Total genomic DNA was extracted from the colon content using the EZNA® Stool DNA Kit (Omega Inc., United States) according to the manufacturer’s instructions. DNA was amplified by using the 341F/805R primer set for the variable region of V3 and V4 (341F: 5′-CCTACGGGNGGCWGCAG-3′, 805R: 5′-GACTACHVGGGTATCTAATCC-3′). Then, PCR products were confirmed with 2% agarose gel electrophoresis. Amplicons were purified using AMPure XT beads (Beckman Coulter Genomics, Danvers, MA, United States) and quantified by Qubit (Invitrogen, United States). Finally, the library was sequenced on a NovaSeq PE250 platform (Illumina, Inc., SanDiego, CA, United States).

The raw data were pre-processed to obtain the high-quality clean tags according to the fqtrim (v0.94). Chimeric sequences were filtered using the Vsearch (v2.3.4). After being filtered, the reads were processed by the DADA2 method using the default parameters to produce a set of denoised amplicon sequence variants (ASVs). ASVs were then annotated to the species with the SILVA 138 database. Alpha diversity was assessed using the Chao 1, Shannon, and Simpson indices. Beta diversity was measured using principal coordinate analysis (PCoA) by the distance between groups based on the unweight UniFrac distance and the hierarchical clustering algorithm. Linear discriminant analysis effect size (LEfSe) and random forest analysis were executed to determine the differences in the bacterial taxa at the genus or higher taxonomy levels between groups. The bacterial function was predicted *via* the phylogenetic analysis of community by reconstruction of unobserved states (PICRUSt2). The predicted sequences were aligned to the Kyoto Encyclopedia of Genes and Genomes (KEGG) database.

### Examination of intestinal histology

The colon segments were dehydrated with a graded ethanol series, cleared in xylene, and embedded in paraffin. The paraffin sections were dewaxed routinely, rehydrated in a graded ethanol series, and stained with hematoxylin and eosin (H&E) and periodic acid-Schiff (PAS), respectively. The slides were digitized with Pannoramic MIDI scanner (3DHISTECH; Budapest, Hungary), and the micrographs were read using CaseViewer software (Version 2.4, 3D HISTECH, Budapest, Hungary). Crypt depth was measured and the expression of mucin in goblet cells was observed by PAS staining. The crypt depth and the number of PAS positive cells were evaluated for five different crypts per section.

### Determination of iNOS activity and NO content

The mucosa samples (0.5 g) were homogenized in 4.5 ml cold PBS solution and then centrifuged at 1,280 × *g* for 10 min to collect supernatants. The assay kits were used to detect iNOS activity and NO content following the manufacturer’s instructions (Shanghai Hengyuan Biological Technology Co., Ltd., China). Absorbance values were measured at 450 nm using a microplate reader (Molecular Devices, CA, United States).

### RNA sequencing

RNA sequencing (RNA-seq) was performed on three biological replicates in each group. Total RNA was extracted using the animal tissue RNA purification kit (LC Science, Houston, TX, United States). The total RNA samples were analyzed by RNA-seq (LC-Bio, China) based on the manufacturer’s protocols. Briefly, poly (A)-containing mRNA molecules were purified from total RNA using poly (T) oligo-attached magnetic beads using two rounds of purification. The purified mRNA was fragmented and reversely transcribed to create the final cDNA library using the mRNA-seq sample preparation kit (Illumina, San Diego, CA, United States). The cDNA libraries were then sequenced using the Illumina sequencing technology on an Illumina Hiseq 4000 system (Illumina, San Diego, CA, United States).

The differentially expressed genes (DEGs) were selected with |log2 fold change (FC)| ≥ 1.0 and *p* < 0.05 using Ballgown R package (R Studio, Inc., Boston, MA, United States). All DEGs were subject to Gene Ontology (GO) enrichment analysis^1^ and KEGG enrichment analysis.^2^

### Real-time quantitative PCR

Real-time quantitative PCR (RT-qPCR) was conducted on eight biological replicates in each group. The mucosa samples (0.1 g) were used for total RNA isolation with TRIzol reagent (15,596,026; Invitrogen, Carlsbad, CA, United States). RNA integrity was verified with 1.1% agarose gel electrophoresis. RNA quantity and quality were determined through detecting optical density at 260 and 280 nm using a micro-spectrophotometer (Hangzhou Allsheng Instruments Co., Ltd., China). RT-PCR was performed with a RT kit (BL699A; Biosharp, China) using a thermal cycler (Bio-Rad, Hercules, CA, United States). A 20 μl reaction mixture consisted of 1 μg of total RNA, 4 μl of RT MasterMix, 1 μl of 20 × Oligo dT & Random Primer, and RNase-free water to bring the final volume to 20 μl. The RT-PCR procedure consisted of 25°C for 10 min, 55°C for 60 min, and 85°C for 5 min. Obtained cDNA was diluted into 10 ng/μl by adding RNase-free water.

The primers were synthesized by Sangon Biotech Co., Ltd. (Shanghai, China), as shown in Supplementary Table 2. RT-qPCR was performed on a LightCycler® 480 System (Roche Diagnostics, Mannheim, Germany). PCR amplification was carried out at a final volume of 20 μl containing 20 ng of cDNA template, 10 μl of FastStart Universal SYBR Green Master (ROX; Roche Applied Science, Mannheim, Germany), 0.06 μl of each primer (100 μM), and RNase-free water to bring the final volume to 20 μl. PCR amplification was performed using the following conditions: 95°C for 1 min, followed by 40 cycles of 95°C for 15 s, and 60°C for 1 min. The geNorm^3^ was used to select the most optimal reference gene by calculating the ranking of three candidate housekeeping genes β-actin, GAPDH, and 18 s (Vandesompele et al., 2002). The most stable candidate housekeeping gene β-actin was selected as the reference gene. Expression levels were determined by the 2^–△△Ct^ method (Livak and Schmittgen, 2001), accounting for gene-specific efficiencies and normalized to β-actin.

### Western blot

Western blot was carried out using eight biological replicates in each group. Total protein was extracted with the lysis buffer for Western and IP (Biosharp, Beijing, China) plus protease inhibitor (Beyotime, Beijing, China) and PhosSTOP (Roche, Basel, Switzerland). The protein concentration was determined with the enhanced BCA protein assay kit (Beyotime, China). 20 μg of total protein was separated on 10 and 12% SDS-PAGE gel and transferred to nitrocellulose membranes. The membranes were blocked with 5% BSA and then incubated with primary antibodies. The primary antibodies used in this experiment were listed in Supplementary Table 3. The membranes were incubated with a horse-radish peroxidase (HRP) conjugated secondary antibody against rabbit IgG at 1:8,000 dilution (Beijing Zhongshan Golden Bridge Biotechnology Co., Ltd. Beijing, China). The protein bands were visualized using an Amersham Imager 600 RGB (General Electric Company, United States) after being treated with an enhanced chemiluminescence (ECL) reagent (Beyotime, China). Obtained bands were quantified using Image J (Version 1.8.0, Rasband, MD, United States). Relative band intensities of target genes were normalized to β-actin expression.

### Immunohistochemistry

Paraffin-embedded tissues were used to detect ZO-1 expression. The paraffin-embedded tissues were cut into 5-μm thick sections, deparaffinized with xylene, and rehydrated in a graded ethanol series. Antigen retrieval was performed by placing the sections in a repair box filled with citric acid, boiling them in a microwave, and then cooling to room temperature. Blocking endogenous peroxidase activity was performed by placing the sections in 3% H_2_O_2_ at room temperature for 25 min. The sections were incubated with mouse anti-pig ZO-1 antibody at 1:500 dilution (Servicebio, China) at 4°C overnight and followed by incubation with a HRP-labeled secondary antibody at 1:1,000 dilution at room temperature for 50 min. The sections stained were visualized with a microscope (Nikon, Tokyo, Japan).

### Statistical analysis

Statistical analyses were performed using SPSS (Version 20.0, SPSS Inc., Chicago, United States). Normality was assessed using the Shapiro–Wilk test and variance homogeneity was tested using Bartlett’s test. Differences between groups were analyzed using one-way ANOVA followed by Tukey’s *post hoc* test. The correlation between the mean diarrhea incidences and the ADG was examined using the Pearson correlation method. Heatmap was generated with pheatmap R package (R Studio, Inc., Boston, MA, United States).

## Results

### Growth performance

The BW and ADG of piglets are shown in Table 1. The BW in the AR21 group was significantly lower than that in the CON group on day 28 (*p* < 0.05) and day 35 (*p* < 0.01). And the BW in the AR7 group was significantly lower than the CON group on days 14, 21, 28, and 35 (*p* < 0.01). Besides, the ADG in the AR21 group was significantly decreased (*p* < 0.01) compared with the CON group from day 21 to day 28, day 28 to day 35, and day 0 to day 35. The ADG in the AR7 group was significantly decreased (*p* < 0.01) compared with the CON group from day 7 to day 14, day 21 to day 28, day 28 to day 35, and day 0 to day 35.

**Table 1:** Effect of artificial rearing on growth performance of piglets.

Items	Treatment			SEM	*p*-Value
	CON	AR21	AR7		
BW (g)^1^					
day 0	1,215	1,242	1,292	19.0	0.248
day 7	2,173	2,205	2,225	31.0	0.802
day 14	3,402	3,379	2,860^**^	60.1	<0.001
day 21	4,727	4,666	3,986^**^	89.0	<0.001
day 28	6,616	5,950^*^	5,385^**^	139.7	<0.001
day 35	9,133	7,550^**^	7,111^**^	218.2	<0.001
ADG (g/day)^2^					
day 0 to day 7	136.9	137.5	133.2	3.33	0.859
day 7 to day 14	175.6	167.8	90.8^**^	7.55	<0.001
day 14 to day 21	189.2	183.8	160.8	5.60	0.085
day 21 to day 28	269.9	183.5^**^	199.8^**^	10.08	<0.001
day 28 to day 35	359.7	228.6^**^	246.6^**^	13.32	<0.001
day 0 to day 35	226.2	180.2^**^	166.2^**^	6.19	<0.001

1 *n* = 10 litters.

2 *n* = 10 litters.

* means *p* < 0.05 compared with the CON group.

** means *p* < 0.01 compared with the CON group.

### Diarrhea occurrence

**Figure 1:**
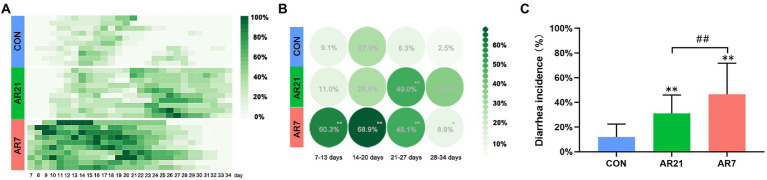
Effects of artificial rearing on the diarrhea incidence in piglets. **(A)** Recorded diarrhea occurrence of piglets from day 7 to day 34. **(B)** Mean diarrhea incidences of piglets for each week. **(C)** Mean diarrhea incidences of piglets from day 7 to day 34. Data are presented with mean ± SD. ^**^p < 0.01 versus the CON group; ^##^*p* < 0.01 versus the AR21 group. *n* = 10 litters.

During the experiment period, the diarrhea occurrence of piglets was recorded and the data are shown in Figures 1A,B. In the AR21 group, the diarrhea incidence was significantly higher (*p* < 0.01) than that in the CON group from day 21 to day 27 and from day 28 to day 34. In the AR7 group, the diarrhea incidence was significantly higher (*p* < 0.05) than that in the CON group at all four periods. The mean diarrhea incidence from day 7 to day 34 was calculated (Figure 1C). To sum up, the diarrhea incidence in the AR7 group was the highest (46.5%); followed by AR21 group (31.1%) and CON group (11.9%). In addition, Pearson correlation analysis was performed between the mean diarrhea incidence and the ADG for each week. The correlation coefficient was −0.645 (*p* < 0.05), indicating a strong negative correlation.

### Relative abundance and diversity of colonic microbiota in piglets

**Figure 2:**
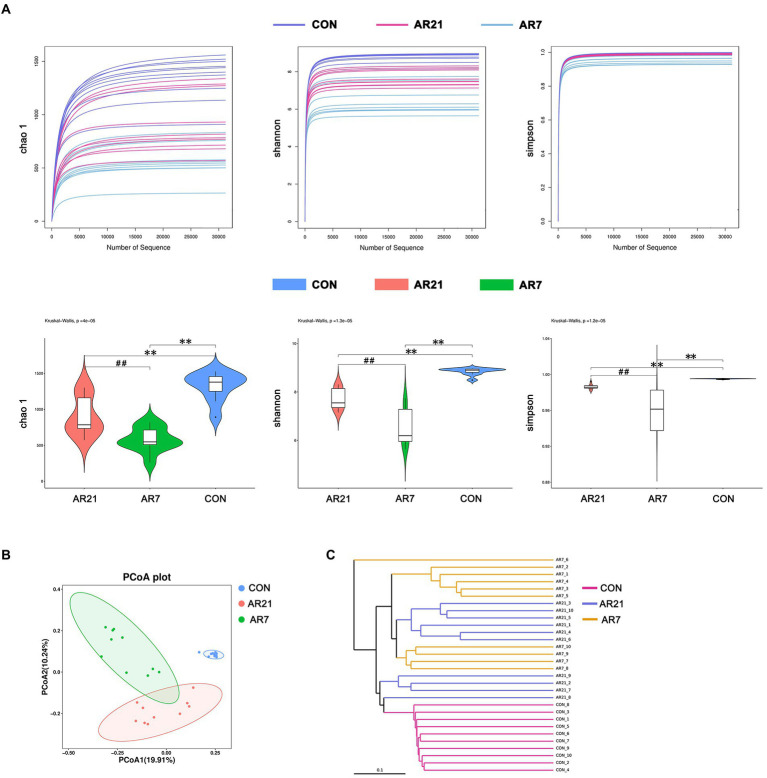
Effects of artificial rearing on the abundance and diversity of colonic microbiota in piglets. **(A)** The alpha diversity determined by Chao 1, Shannon, and Simpson. **(B)** The principal coordinate analysis (PCoA) score based on the unweight UniFrac distance. **(C)** UPGMA hierarchical clustering analysis based on the unweighted UniFrac distance. Data are presented with mean ± SD. ^**^p < 0.01 versus the CON group; ^##^*p* < 0.01 versus the AR21 group. *n* = 10 piglets.

As shown in Figure 2A, the alpha diversity of microbial community was evaluated using Chao 1 index, Shannon index, and Simpson index, respectively. The Chao 1, Shannon, and Simpson indices in the AR7 and AR21 groups were significantly lower (*p* < 0.05) than those in the CON group, indicating that artificial rearing reduced the microbial abundance and diversity. PCoA analysis based on the unweight UniFrac distance revealed a significant difference in beta diversity of microbial community between three groups (Figure 2B). The results of hierarchical cluster analysis by UPGMA (Figure 2C) showed that all the samples were divided into three clusters according to different treatments and the level of the AR21 group was closer to that of the AR7 group.

**Figure 3:**
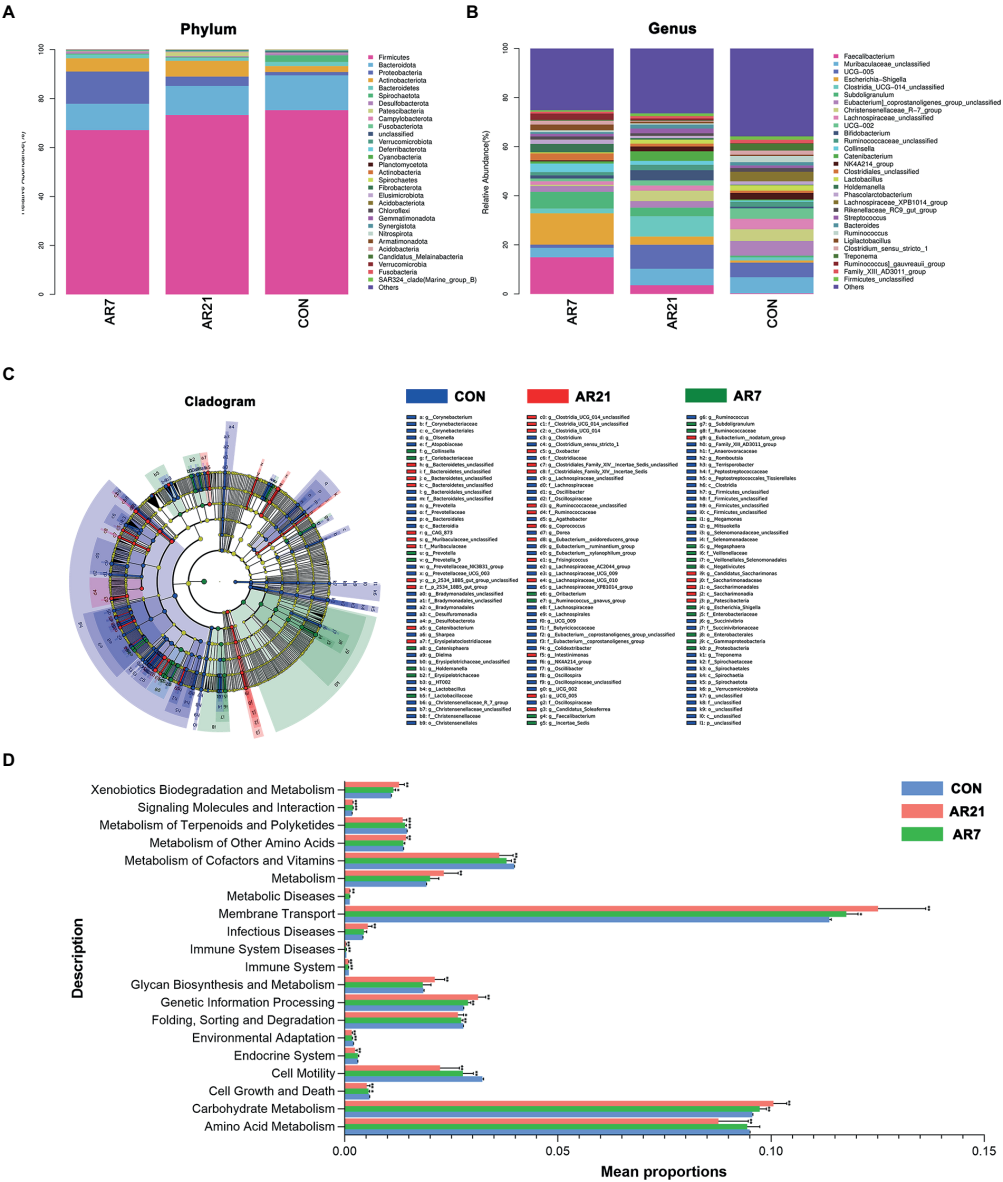
Effects of artificial rearing on the structure and function of colonic microbiota in piglets. **(A)** Relative abundance of microbiota at the phylum level. **(B)** Relative abundance of microbiota at the genus level. **(C)** Cladogram of enriched taxa based on linear discriminant analysis effect size (LEfSe) analysis. **(D)** Differences in bacterial function at Kyoto Encyclopedia of Genes and Genomes (KEGG) level 2 using PICRUSt2. Data are presented with mean ± SD. ^*^p < 0.05 versus the CON group; ^**^p < 0.01 versus the CON group. *n* = 10 piglets.

Figures 3A,B showed the composition of microbial community at the phylum and genus levels. *Muribaculaceae* (6.56%) was the most abundant genera in the CON group, followed by *Eubacterium coprostanoligenes group* (5.96%) and *Ruminococcaceae* UCG-005 (5.95%). In the AR21 group, the three most abundant genera were *Ruminococcaceae* UCG-005 (9.77%), *Clostridia* UCG-014 (8.28%), and *Muribaculaceae* (6.72%). In the AR7 group, *Faecalibacterium* (14.94%) was the most abundant genera, followed by *Escherichia-Shigella* (12.78%), and *Subdoligranulum* (6.82%). LEfSe analysis (Figure 3C) further indicated the relative abundance of *Catenibacterium*, *Clostridia* UCG-014, *Ruminococcaceae*, *Bacteroidetes*, and *Muribaculaceae* showed an upward trend (*p* < 0.05) in the AR21 group. There was a significant increase (*p* < 0.05) in the relative abundance of *Holdemanella*, *Megasphaera*, *Escherichia Shigella*, *Collinsella*, and *Prevotella* in the AR7 group. The potential functions of microbial community were predicted by the PICRUSt2 (Figure 3D). At KEGG level 2, carbohydrate metabolism, membrane transport, and xenobiotics biodegradation and metabolism were significantly increased (*p* < 0.05) in AR7 and AR21 groups compared to the CON group. Cell motility, environmental adaptation, immune system, metabolism of cofactors and vitamins, metabolism of terpenoids and polyketides, signaling molecules and interaction, and folding, sorting, and degradation were significantly decreased (*p* < 0.05) in AR7 and AR21 groups compared to the CON group.

### Histology in piglet colon

**Figure 4:**
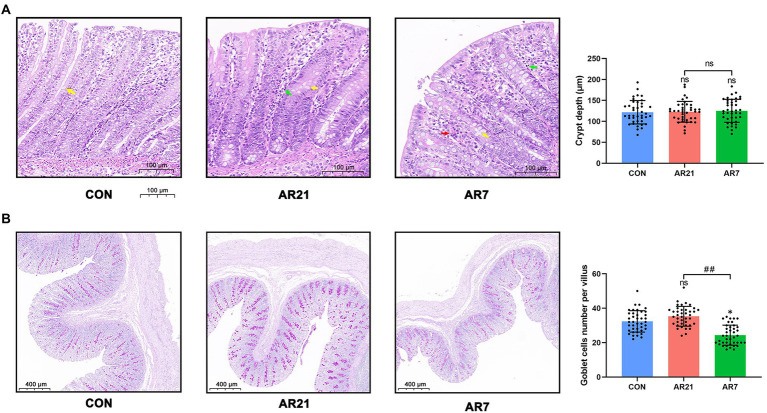
Effects of artificial rearing on the histology in piglet colon. **(A)** Microstructure of piglet colon (H&E staining). Yellow arrow indicates vacuoles. Green arrow indicates leukocyte infiltration. Red arrow indicates red blood cell infiltration. **(B)** Mean number of goblet cells (PAS staining). Data are presented with mean ± SD. ^ns^*p* > 0.05; ^*^p < 0.05 versus the CON group; ^##^*p* < 0.01 versus the AR21 group. *n* = 8 piglets.

To evaluate the effects of artificial rearing on intestinal morphology, we observed the microstructure of colon stained with H&E (Figure 4A). The colon tissue from the CON piglets displayed the absence of inflammatory cells and normal villous architecture with orderly arrangement of intestinal epithelial cells, long and slender villus, as well as smooth villus border. In the AR21 group, we observed leukocyte infiltration (green arrow) in the mucosa. In the AR7 group, the colon tissue revealed the infiltrations of red blood cells (red arrow) and leukocytes (green arrow) in the mucosa. Given the role of mucin in maintaining protective mucus barriers, the goblet cells were stained with PAS and then counted (Figure 4B). There was a significant reduction (*p* < 0.05) of the number of goblet cells in the AR7 group when compared with the CON group.

### Mucosal barrier function in piglet colon

**Figure 5:**
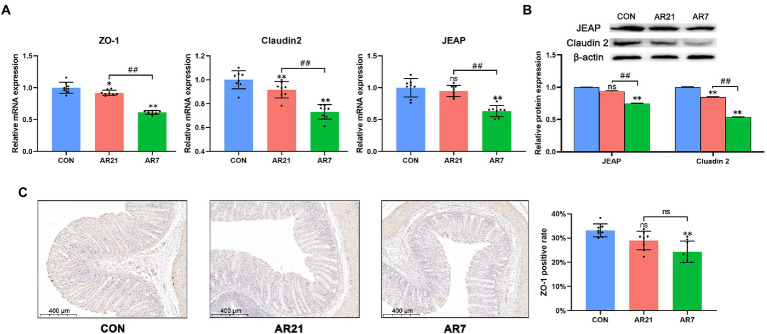
Effects of artificial rearing on the tight junction proteins in piglet colon. **(A)** Relative mRNA expression of tight junction proteins. **(B)** Relative protein expression of tight junction proteins. **(C)** ZO-1 positive cells by IHC staining. Data are presented with mean ± SD. ^ns^*p* > 0.05; ^*^p < 0.05 versus the CON group; ^**^p < 0.01 versus the CON group; ^##^*p* < 0.01 versus the AR21 group. *n* = 8 piglets.

Then, we detected the mRNA and protein expression of tight junction proteins including ZO-1, Claudin-2, and JEAP (Figure 5). In the AR21 group, the mRNA expression of ZO-1 and Claudin-2 was lower (*p* < 0.05) than the CON group. And the protein expression of Claudin-2 in the AR21 group was lower (*p* < 0.05) than the CON group. In the AR7 group, the mRNA and protein expression of ZO-1, Claudin-2, and JEAP was significantly lower (*p* < 0.01) than that in the CON group.

### DEGs, KEGG, and GO enrichment analysis

**Figure 6:**
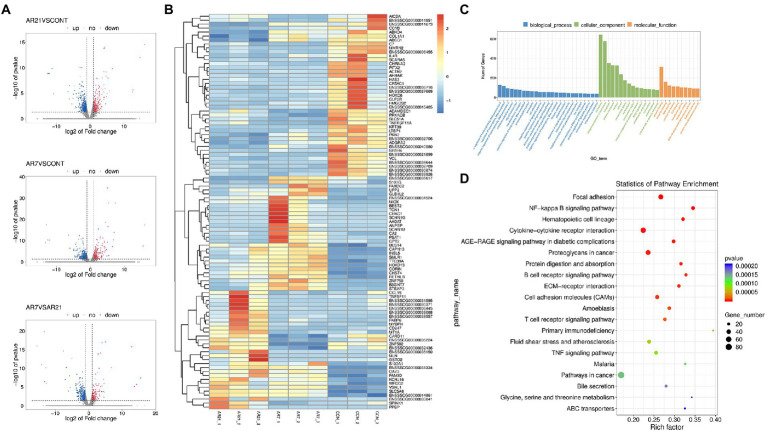
Effects of artificial rearing on the transcriptome profile in piglet colon. **(A)** The volcano plot for the DEGs. Blue dots mean the up-regulated genes. Red dots mean the down-regulated genes. Grey dots mean the genes with no significant changes. **(B)** Heatmap of DEGs in RNA-seq. Relative expression of DEGs where red hues represent comparatively upregulated genes and blue hues represent comparatively downregulated genes (scale at right). **(C)** GO enrichment analysis of DEGs. **(D)** KEGG pathway enrichment analysis of DEGs. *n* = 3 piglets.

Genes conformed to false discovery rate (FDR) < 0.05 and |log2 fold change (FC)| ≥ 1.0 were considered to be DEGs between two groups. As shown in Figures 6A,B, 868 DEGs were identified between the AR21 group and the CON group, including 281 upregulated DEGs and 587 downregulated DEGs. And 833 DEGs were identified between the AR7 group and the CON group, including 231 upregulated DEGs and 602 downregulated DEGs.

As shown in Figure 6C, the DEGs were annotated with the GO enrichment analysis, with one or more GO terms, including biological process (840 subclasses), cellular component (96 subclasses), and molecular function (172 subclasses). For biological process, the major categories represented were positive regulation of transcription by RNA polymerase II (GO:0045944) and regulation of transcription, DNA-templated (GO:0006355). For the cellular components, the major categories were membrane (GO:0016020) and integral component of membrane (GO:0016021). For the molecular function, the major categories were protein binding (GO:0005515) and metal ion binding (GO:0046872). A number of interesting categories were also obtained, such as oxidation–reduction process (GO:0055114), immune response (GO:0006955), and inflammatory response (GO:0006954), which may play a role in artificial rearing-caused diarrhea and colon injury.

The DEGs were further annotated with the KEGG pathway analysis and classified into 86 subclasses. Figure 6D illustrated the scatterplot of KEGG enrichment analysis. The four most enriched pathways were Focal adhesion (ko04510), NF-κB signaling pathway (ko04064), Hematopoietic cell lineage (ko04640), and Cytokine-cytokine receptor interaction (ko04060). A number of interesting pathways were also obtained, such as bile secretion (ko04976), mineral absorption (ko04978), and carbohydrate digestion and absorption (ko04973), which is closely related to the digestion and absorption of nutrients.

### NF-κB signaling pathway

**Figure 7:**
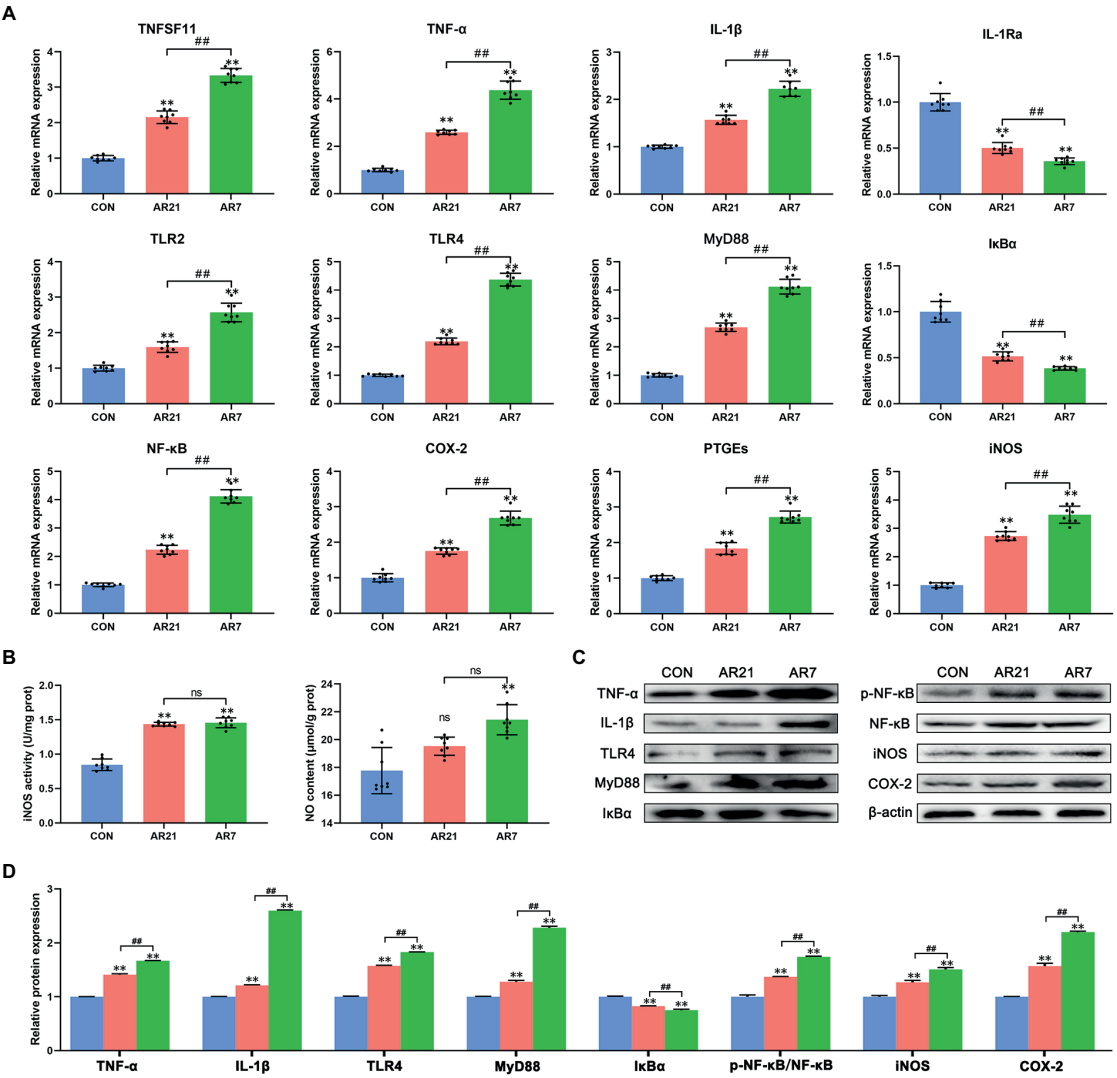
Effects of artificial rearing on NF-κB pathway and its downstream genes in piglet colon. **(A)** Relative mRNA expression of NF-κB pathway genes. **(B)** iNOS activity and NO content. **(C,D)** Relative expression of NF-κB pathway proteins and it downstream proteins. Data are presented with mean ± SD. ^ns^*p* > 0.05; ^**^p < 0.01 versus the CON group; ^##^*p* < 0.01 versus the AR21 group. *n* = 8 piglets.

NF-κB signaling pathway (ko04064) was found to be highly enriched and the DEGs were verified by RT-qPCR and western blot, including TNFSF11, TNF (TNF-α), IL-1Ra, TLR2, NFKBIA (IκBα), and PTGS2 (COX-2). As presented in Figure 7A, compared with the CON group, the mRNA expression of TNFSF11, TNF-α, IL-1β, TLR2, TLR4, MyD88, NF-κB (p65), COX-2, PTGEs, and iNOS was significantly increased (*p* < 0.01), whereas the mRNA expression of IL-1Ra and IκBα was significantly decreased (*p* < 0.01) in AR21 and AR7 groups. Besides, iNOS activity and NO content were detected using the assay kits (Figure 7B). There were significant increases in iNOS activity in AR21 and AR7 groups (*p* < 0.01) as well as NO content in the AR7 group (*p* < 0.01) when compared to the CON group. These results indicated an activation of NF-κB pathway, which could result in the production of cytokines and thereby promote inflammatory response. As expected, the protein expression was indeed consistent with their corresponding transcription status (Figures 7C,D).

### Cytokine secretion

**Figure 8:**
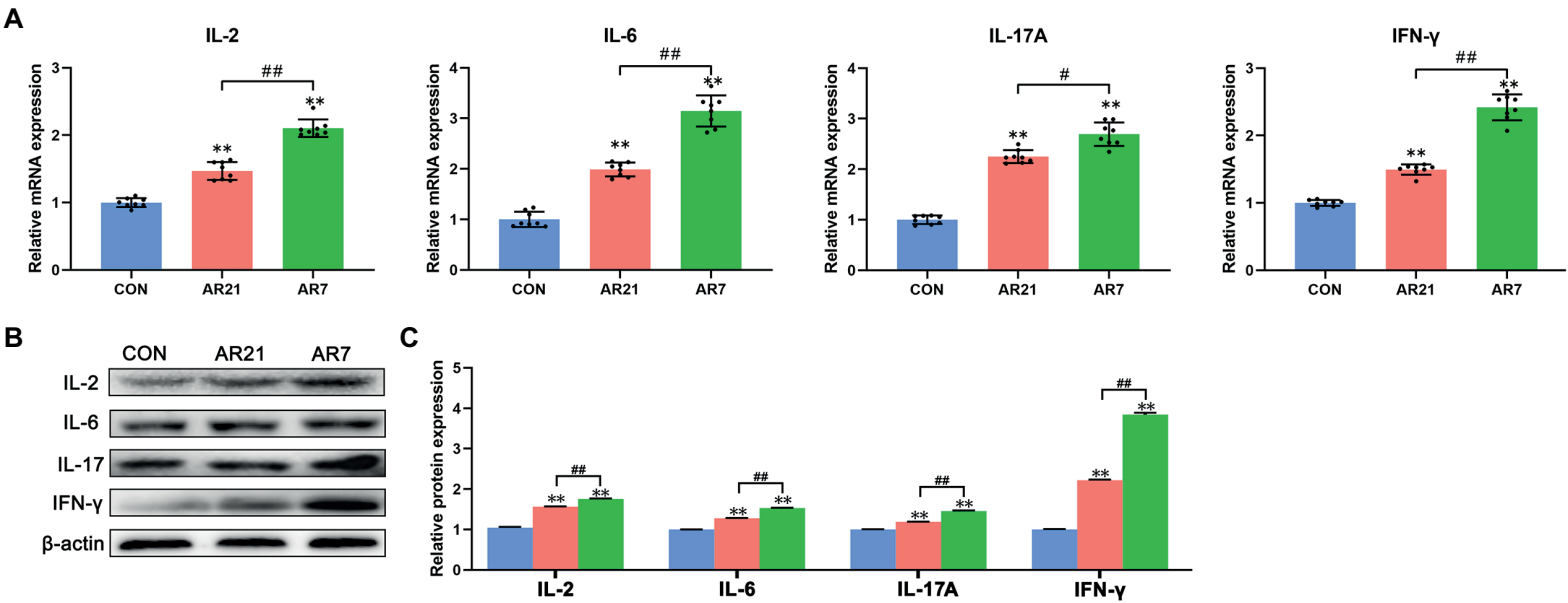
Effects of artificial rearing on cytokines in piglet colon. **(A)** Relative mRNA expression of IL-2, IL-6, IL-17A, and IFN-γ. **(B,C)** Relative protein expression of IL-2, IL-6, IL-17A, and IFN-γ. Data are presented with mean ± SD. ^**^p < 0.01 versus the CON group; ^##^*p* < 0.01 versus the AR21 group. *n* = 8 piglets.

Cytokine-cytokine receptor interaction (ko04060) was also found to be highly enriched and the DEGs were verified by RT-qPCR and western blot, including IL-2, IL-6, IL-17A, and IFN-γ (Figure 8). In the AR21 and AR7 groups, relative mRNA and protein expression of IL-2, IL-6, IL-17A, and IFN-γ were significantly higher (*p* < 0.01) than those in the CON group. In the AR7 group, relative mRNA and protein expression of the detected cytokines were significantly higher (*p* < 0.01) than those in the AR21 group.

### SLC family

**Figure 9:**
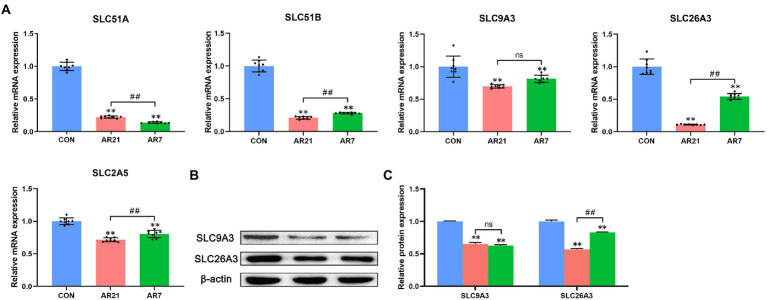
Effects of artificial rearing on SLC family members in piglet colon. **(A)** Relative mRNA expression of SLC51A, SLC51B, SLC9A3, SLC26A3, and SLC2A5. **(B,C)** Relative protein expression of SLC9A3 and SLC26A3. Data are presented with mean ± SD. ^ns^*p* > 0.05; ^**^p < 0.01 versus the CON group; ^#^*p* < 0.05 versus the AR21 group; ^##^*p* < 0.01 versus the AR21 group. *n* = 8 piglets.

To investigate the mechanism of artificial rearing-induced diarrhea and colon injury, eight DEGs were verified by RT-qPCR, which was related to the digestion and absorption of nutrients. As presented in Figure 9, all detected SLC family members were significantly downregulated (*p* < 0.01) in the AR21 and AR7 groups compared with the CON group. It is noteworthy that the mRNA expression of SLC51B, SLC2A5, and SLC26A3 in the AR7 group was significantly higher (*p* < 0.01) than that in the AR21 group. The protein expression of SLC9A3 and SLC26A3 was indeed consistent with their corresponding transcription status.

In addition, the expression patterns of the 11 DEGs were generally consistent with the expression patterns identified by RNA-seq, suggesting that the RNA-seq results were accurate and reliable (Supplementary Table 4).

## Discussion

Previous studies have shown the adverse effects of early weaning on the growth performance of pigs (Leliveld et al., 2013; Ming et al., 2021). Similar to early weaning, artificial rearing also brought about a number of stressors, such as an abrupt separation from the sow and a different food source (Baxter et al., 2013). Schmitt et al. (2019) found that artificial rearing from 7 days of age reduced weaning BW of piglets. In this study, both AR21 and AR7 piglets had lower weaning weight and ADG during the entire experimental period compared with the CON piglets. Furthermore, the pre-weaning diarrhea incidence in AR7 group (46.5%) was higher than that in the AR21 group (31.1%) and the CON group (11.9%) during the entire experimental period, while there was a strong negative correlation between ADG and diarrhea incidence. These results indicated that artificial rearing increased the incidence of diarrhea and thus reduced growth performance in piglets.

The establishment of a healthy intestinal microbiota is required in maintaining intestinal homeostasis in the newborn, a time that is critical for immunological development (Koleva et al., 2015). The diversity of intestinal microbiota is an indicator for evaluating microbiota’s health status. According to the alpha diversity analysis of 16S rDNA sequencing results, the microbial abundance and diversity were the highest in the CON group, followed by AR21 group and then AR7 group. Meanwhile, it was found by PCoA score that the difference in microbial community was obvious between three groups. This showed that artificial rearing reduced the microbial diversity and altered the structure of colonic microbiota, which is consistent with several studies reporting that artificial rearing reduced the abundance and diversity of ruminal microbiota in lambs (Belanche et al., 2019; Huang et al., 2022).

Moreover, this study also revealed the effects of artificial rearing on the composition of microbial community at the genus level. We noticed that *Ruminococcaceae* UCG-005, *Catenibacterium*, and *Muribaculaceae* increased significantly in the AR21 group. *Faecalibacterium*, *Escherichia-Shigella*, *Subdoligranulum*, and *Prevotella* increased significantly in the AR7 group. Fu et al. (2021) reported that the rise in *Ruminococcaceae* UCG-005 was correlated with intestinal inflammation and injury in piglets. *Muribaculaceae* was capable of degrading carbohydrates (Wang et al., 2020). *Catenibacterium*, *Faecalibacterium*, *Subdoligranulum*, and *Prevotella* were typical post-weaning microbial groups, which was associated with the introduction of non-breast foods and the digestion of dietary fibers (Kageyama and Benno, 2000; Luu et al., 2019; Parada Venegas et al., 2019; Van Hul et al., 2020). The genus of *Escherichia-Shigella* was a gram-negative pathogenic bacterium, which was responsible for Crohn’s disease (Pascal et al., 2017). In addition, the prediction of bacterial function showed that artificial rearing changed a wide range of pathways, such as carbohydrate metabolism, xenobiotics biodegradation and metabolism, environmental adaptation, and immune system. Similar results were also reported in two other studies that formula feeding promoted carbohydrate metabolism in infant feces (Edwards et al., 1994; Bridgman et al., 2017). Based on these results, we concluded that artificial rearing could increase relative abundance of harmful bacteria and influence metabolic processes, which might led to the diarrhea of piglets.

The mucosa is the first line of defense against invading pathogens, which is composed of colonocytes and goblet cells in colon. The goblet cells are responsible for mucus secretion that form a protective mucus layer (Birchenough et al., 2015). Clinical data indicated a strong link between goblet cell loss and the development of ulcerative colitis (Gersemann et al., 2009). Besides, the spaces between colonocytes are sealed by tight junction proteins, including ZO proteins and Claudins, which provides a physical barrier to selectively allow nutrient absorption while excluding bacteria (Buckley and Turner, 2018). Prior work reported that artificial rearing from 7 days of age caused villus atrophy and deeper crypt in the small intestine of piglets (Vergauwen et al., 2017). In rats, infant formula feeding led to stunted villus and decreased the expression of ZO-1, Claudin-3, and Claudin-4 in the ileum (Gong et al., 2020). Singh et al. (2020) found that formula feeding resulted in villus loss, a decrease in ZO-1, and apoptosis of intestinal epithelial cells in rat ileum. In this study, we observed the infiltrations of red blood cells and leukocytes accompanied by a reduction of goblet cells and the decreases of ZO-1, Claudin-2 and JEAP in the AR7 group. It can thus be concluded that artificial rearing from 7 days of age caused morphology disruption and disrupted barrier function in colonic mucosa. It is noteworthy that artificial rearing from 21 days of age had no significant effects on the number of goblet cells and the expression of ZO-1 and JEAP. These findings supported the notion that earlier weaning caused more serious physiological changes in structure and function of intestine (Pluske et al., 1997; Levast et al., 2010; Campbell et al., 2013).

NF-κB signaling pathway plays an essential role in immune response and inflammatory process. TLRs mediate the activation of NF-κB through the recognition of pathogen-associated molecular patterns (PAMPs) present on various microbes (Duncan et al., 2017). As the adapters for TLRs and IL-1R, MyD88 can lead to IκBα degradation and NF-κB nuclear translocation, which initiates the transcription and production of pro-inflammatory cytokines and chemokines (Kawasaki and Kawai, 2014). Previous study reported that the upregulation of TLR4, MyD88, and NF-κB was involved in lipopolysaccharide (LPS)-induced inflammatory injury through the enhanced production of pro-inflammatory cytokines including IL-1β, TNF-α, and IL-6 in the colonic mucosa of piglets (Gao et al., 2021). Tang et al. (2021) found that early weaning induced inflammation and impaired intestinal barrier function through the upregulation of TLR4, IL-1β, IL-6, and TNF-α in piglet colon. Pie et al. (2004) also reported that weaning increased the expression of IL-1β, IL-6, and TNF-α in the mid-small intestine and up-regulated the expression of IL-1β in the proximal colon of piglets. In line with these findings, artificial rearing from 21 and 7 days of age induced colonic inflammation through the activation of NF-κB pathway and the induction of pro-inflammatory cytokines in piglets.

The SLC family constitutes a group of membrane transport proteins that transports diverse solutes across biological membranes and participates in many physiological functions, including nutrient absorption, metabolic transformation, energy homeostasis, and host defense (Lin et al., 2015). For instance, SLC51A, SLC51B, and SLC9A3 are involved in bile acid reclamation and bile secretion. Song et al. (2021) reported that the expression of SLC9A3 was decreased in the small intestine of piglets after porcine epidemic diarrhea virus infection and severe diarrhea occurred. SLC2A5 is a fructose transporter and engages in the digestion and absorption of complex carbohydrates (Iizuka, 2017). Deoxynivalenol contamination reduced SLC2A5 expression in chicken jejunum and led to growth depression (Dietrich et al., 2012). SLC26A3 is a key Cl^−^/HCO_3_^−^ exchanger protein and the patients with inflammatory bowel disease (IBD) showed a reduction in SLC26A3 in colonic epithelial cells compared with the healthy controls (Farkas et al., 2011). In the present study, the mRNA expression of SLC51A, SLC51B, SLC9A3, SLC2A5, and SLC26A3 decreased in the AR7 group. These observations indicated that the dysfunction of SLC family affected solute transport and destroyed intestinal homeostasis, contributing to diarrhea. Notably, the mRNA expression of SLC51B, SLC2A5, and SLC26A3 in the AR21 group was lower than that in the AR7 group, which might explain a higher diarrhea incidence in the AR21 group from days 28 to 34.

## Concluding remarks

Collectively, our results indicated that artificial rearing both from 21 and 7 days of age reduced growth performance and increased diarrhea incidence in piglets. In addition, artificial rearing changed the diversity, structure, and function of microbial community in piglets. Meanwhile, artificial rearing mediated inflammatory response and affected solute transport through the dysfunction of SLC family.

## Data availability statement

The datasets presented in this study can be found in online repositories. The names of the repository/repositories and accession number(s) can be found at: http://www.ncbi.nlm.nih.gov/bioproject/, 855325; http://www.ncbi.nlm.nih.gov/bioproject/, 855587.

## Ethics statement

The animal study was reviewed and approved by Institutional Animal Care and Use Committee of Northeast Agricultural University.

## Author contributions

JB, HL, XL, and RZ made substantial contributions to the conception or design of the work. QH conducted data analysis and drafted the work. JB, XZ, and HN revised the work critically for important intellectual content. All authors contributed to the article and approved the submitted version.

## Funding

The study was supported by the China Agriculture Research System of MOF and MARA (No. CARS-35-05B).

## Conflict of interest

The authors declare that the research was conducted in the absence of any commercial or financial relationships that could be construed as a potential conflict of interest.

## Publisher’s note

All claims expressed in this article are solely those of the authors and do not necessarily represent those of their affiliated organizations, or those of the publisher, the editors and the reviewers. Any product that may be evaluated in this article, or claim that may be made by its manufacturer, is not guaranteed or endorsed by the publisher.

## Footnotes

^1^ http://geneontology.org/

^2^ https://www.genome.jp/kegg/

^3^ http://medgen.ugent.be/∼jvdesomp/genorm/
