# Supplementary material for: Artificial rearing alters intestinal microbiota and induces inflammatory response in piglets
Source: Front Microbiol. 2022 Oct 6;13:1002738. doi: 10.3389/fmicb.2022.1002738 (PMC9584613; doi:10.3389/fmicb.2022.1002738)
Supplement: Supplementary file 1 [file Table_1.docx]

# Supplementary Tables

## Supplementary Table 1

**Supplementary Table 1** *Nutrient composition of the milk replacer*

| Nutrient composition | Values^1^ |
| --- | --- |
| Crude protein (%) | 19.80 |
| Crude ash (%) | 9.90 |
| Crude fibre (%) | 1.00 |
| Crude fat (%) | 12.80 |
| Methionine (%) | 0.54 |
| Lysine (%) | 1.49 |
| Calcium (%) | 0.60 |
| Peroxide value (meq/kg) | 15.0 |
| Sodium chloride (%) | 1.0 |
| Lactose (%) | 39.0 |
| Moisture (%) | 5.0 |
| Phosphrous (%) | 0.5 |
| Acid value (mg(KOH)/g) | 15.0 |

^1^The nutrient levels are guaranteed analytical values.

## Supplementary Table 2

**Supplementary Table 2** Primers used for qRT-PCR

| Gene | NCBI ID | Primer | |
| --- | --- | --- | --- |
| ZO-1 | XM_021098856.1 | Forward5’-CCAGGGAGAGAAGTGCCAGTAGG-3’ | |
|  |  | Reverse 5’-TTTGGTGGGTTTGGTGGGTTGAC-3’ | |
| Claudin 2 | XM_021079578.1 | Forward 5’-CGCTCCAACTACTACGATGCCTATC-3’ | |
|  |  | Reverse 5’-ACTCTTGGCTTTGGGTGGTTGAC-3’ | |
| JEAP | XM_005667237.3 | Forward 5’-GGACACCACGATCATCAACCACTC-3’ | |
|  |  | Reverse 5’-CCTCCTCCTCCTGCCAGATGTG-3’ | |
| TNFSF11 | XM_001925694.6 | Forward 5’-GTTCCCACAAAGTGAGTCTGTCCTG-3’ | |
|  |  | Reverse 5’-TCCCGTTGCTGAAAGTCATGTTGG-3’ | |
| TNF-α | NM_214022.1 | Forward 5’-GCACTGAGAGCATGATCCGAGAC-3’ | |
|  |  | Reverse 5’-CGACCAGGAGGAAGGAGAAGAGG-3’ | |
| IL-1β | NM_001302388.2 | Forward 5’-CAAGCCAGAGAAGCAAGGTGTCC-3’ | |
|  |  | Reverse 5’-GCCGTCCTCAGCAGCAAGAAG-3’ | |
| IL-1Ra | NM_214262.1 | Forward 5’-GCTGTGCCTGTCCTGTGTCAAG-3’ | |
|  |  | Reverse 5’-TTGTCCTGCTCGCTGTTCTTTCTC-3’ | |
| TLR2 | NM_213761.1 | Forward 5’-ACTACTCCAACCCTTCCGGT-3’ | |
|  |  | Reverse 5’-TGATGAATGAACGCAGCCCA-3’ | |
| TLR4 | NM_001113039.2 | Forward 5’-GACGAAGACTGGGTGAGGAATGAAC-3’ | |
|  |  | Reverse 5’-CCTGGATGATGTTAGCAGCGATGG-3’ | |
| MyD88 | NM_001099923.1 | Forward 5’-CTGCGTCTGGTCCATTGCTAGTG-3’ | |
|  |  | Reverse 5’-TTCTGATGGGCACCTGGAGAGAG-3’ | |
| IκBα | NM_001005150.1 | Forward 5’-TGGTGTCGCTCTTGTTGAAGTGTG-3’ | |
|  |  | Reverse 5’-GCTGCTGTATCCGAGTGCTTGG-3’ | |
| NF-κB (p65) | NM_001114281.1 | Forward 5’-CTGAGGCTATAACTCGCTTGGTGAC-3’ | |
|  |  | Reverse 5’-CATGTCCGCAATGGAGGAGAAGTC-3’ | |
| COX-2 | NM_214321.1 | Forward 5’-CTCCGACAGCCAAAGACACTCAAG-3’ | |
|  |  | Reverse 5’-TCATCAGACCAGGCACCAGACC-3’ | |
| PTGEs | NM_001038631.1 | Forward 5’-ACGCTGCTGGTCATCAAGATGTAC-3’ | |
|  |  | Reverse 5’-CCTCCGTGTCTCTGAGCATCCTC-3’ | |
| iNOS | NM_001143690.1 | Forward 5’-AGGAAGTGGGCAGAAGGATGGG-3’ | |
|  |  | Reverse 5’-GGAGCACGGCGACATTGATCTC-3’ | |
| IL-2 | NM_213861.1 | Forward 5’-ACAAAGAAACAACTGGAGCCATTGC-3’ | |
|  |  | Reverse 5’-GAGCATCCTGGAGAGATCAGCATTC-3’ | |
| IL-6 | NM_001252429.1 | Forward 5’-AAATGTCGAGGCCGTGCAGATTAG-3’ | |
|  |  | Reverse 5’-GGGTGGTGGCTTTGTCTGGATTC-3’ | |
| IL-17A | NM_001005729.1 | Forward 5’-CAGCAAGCTCCAGCTCATCCATC-3’ | |
|  |  | Reverse 5’-CAGCAGAAGCAGCAGTGACAGG-3’ | |
| IFN-γ | NM_213948.1 | Forward 5’-CGCAAAGCCATCAGTGAACTCATC-3’ | |
|  |  | Reverse 5’-TTTGATGCTCTCTGGCCTTGGAAC-3’ | |
| SLC51A | NM_001244266.1 | Forward 5’-GGTGAGCAGAACATAGGAGCCAAG-3’ | |
|  |  | Reverse 5’-AAGGGAGGCGAACAAGCAATCTG-3’ | |
| SLC51B | XM_005658570.3 | Forward 5’-GTGGCTGTGGTGGTCGTGATAAG-3’ | |
|  |  | Reverse 5’-AGGTAGAGGGCTTCTGGATGCTG-3’ | |
| SLC9A3 | XM_021077062.1 | Forward 5’-CATCTTCTTCACCGTCATCGTCCAG-3’ | |
|  |  | Reverse 5’-CGCTGTTCACTCCTCTTCACCTTC-3’ | |
| SLC26A3 | NM_001130248.1 | Forward 5’-ATGGTGGGAGCAGTAGTGGTGAG-3’ | |
|  |  | Reverse 5’-GCCAGAAAGAATTGTGACCGTTGC-3’ | |
| SLC8A3 | XM_013989127.2 | Forward 5’-TGAAGATGAGTCAGGCGAGGAGAG-3’ | |
|  |  | Reverse 5’-GATGAGGATGGAGACAACGAAGCAG-3’ | |
| SLC3A1 | NM_001123042.1 | Forward 5’-CACCATCTCCATCATCGCCATCTC-3’ | |
|  |  | Reverse 5’-CCCGTCTTTGTCGCTGTCCTTG-3’ | |
| SLC2A5 | XM_021095282.1 | Forward 5’-AGAGCACTCCTGATCCTGTACTTCC-3’ | |
|  |  | Reverse 5’-CAGGTAGCACAGAGCCACAAAGG-3’ | |
| SLC15A1 | NM_214347.1 | Forward 5’-AGAGCACTCCTGATCCTGTACTTCC-3’ | |
|  |  | Reverse 5’-CAGGTAGCACAGAGCCACAAAGG-3’ | |
| β-actin | XM_021086047.1 | Forward 5’-GGCACCACACCTTCTACAACGAG-3’ | |
|  |  | Reverse 5’-TCATCTTCTCACGGTTGGCTTTGG-3’ |  |

## Supplementary Table 3

**Supplementary Table 3** Antibodies used for western blot

| Primary antibodies | Dilution ratio |
| --- | --- |
| JEAP | 1:500 |
| Claudin 2 | 1:2000 |
| TNF-α | 1:1000 |
| IL-1β | 1:500 |
| TLR4 | 1:500 |
| MyD88 | 1:2000 |
| p-NF-κB (p-p65) | 1:500 |
| NF-κB (p65) | 1:2000 |
| iNOS | 1:500 |
| COX-2 | 1:500 |
| IL-2 | 1:750 |
| IL-6 | 1:500 |
| IL-17 | 1:500 |
| IFN-γ | 1:500 |
| β-actin | 1:75000 |

## Supplementary Table 4

**Supplementary Table 4** Validation of RNA-seq gene expression data by qRT-PCR

| Gene | AR21 vs. CON | | AR7 vs. CON | |
| --- | --- | --- | --- | --- |
|  | RNA-seq  (log_2_ FC) | qRT-PCR  (2^-△△CT^) | RNA-seq  (log_2_ FC) | qRT-PCR  (2^-△△CT^) |
| TNFSF11 | 1.459 | 2.161 | 2.319 | 3.340 |
| TNF-α | 0.319 | 2.608 | 0.516 | 4.381 |
| IL-1Ra | -0.384 | 0.502 | -0.610 | 0.358 |
| TLR2 | -0.868 | 1.598 | -1.237 | 2.579 |
| IκBα | -1.120 | 0.518 | -0.495 | 0.387 |
| COX-2 | 0.401 | 1.759 | 1.163 | 2.653 |
| SLC51A | -1.863 | 0.224 | -3.399 | 0.138 |
| SLC51B | -1.854 | 0.212 | -2.157 | 0.283 |
| SLC9A3 | -0.186 | 0.713 | -2.254 | 0.830 |
| SLC2A5 | -0.218 | 0.716 | -2.008 | 0.803 |
| SLC26A3 | -0.431 | 0.108 | 1.008 | 0.554 |
